# HLA variants have different preferences to present proteins with specific molecular functions which are complemented in frequent haplotypes

**DOI:** 10.3389/fimmu.2022.1067463

**Published:** 2022-12-20

**Authors:** Vadim Karnaukhov, Wayne Paes, Isaac B. Woodhouse, Thomas Partridge, Annalisa Nicastri, Simon Brackenridge, Dmitrii Shcherbinin, Dmitry M. Chudakov, Ivan V. Zvyagin, Nicola Ternette, Hashem Koohy, Persephone Borrow, Mikhail Shugay

**Affiliations:** ^1^Center of Life Sciences, Skolkovo Institute of Science and Technology, Moscow, Russia; ^2^Shemyakin-Ovchinnikov Institute of Bioorganic Chemistry, Russian Academy of Science, Moscow, Russia; ^3^Nuffield Department of Clinical Medicine, University of Oxford, Oxford, United Kingdom; ^4^Medical Research Council (MRC) Human Immunology Unit, Medical Research Council (MRC) Weatherall Institute of Molecular Medicine (WIMM), John Radcliffe Hospital, University of Oxford, Oxford, United Kingdom; ^5^Medical Research Council (MRC) Weatherall Institute of Molecular Medicine (WIMM) Centre for Computational Biology, Medical Research Council (MRC) Weatherall Institute of Molecular Medicine (WIMM), University of Oxford, Oxford, United Kingdom; ^6^The Jenner Institute, Nuffield Department of Medicine, University of Oxford, Oxford, United Kingdom; ^7^Center for Precision Genome Editing and Genetic Technologies for Biomedicine, Pirogov Russian National Research Medical University, Moscow, Russia

**Keywords:** HLA, gene ontologies and pathways, molecular function, protein, mass spectrometry

## Abstract

Human leukocyte antigen (HLA) genes are the most polymorphic loci in the human genome and code for proteins that play a key role in guiding adaptive immune responses by presenting foreign and self peptides (ligands) to T cells. Each person carries up to 6 HLA class I variants (maternal and paternal copies of HLA-A, HLA-B and HLA-C genes) and also multiple HLA class II variants, which cumulatively define the landscape of peptides presented to T cells. Each HLA variant has its own repertoire of presented peptides with a certain sequence motif which is mainly defined by peptide anchor residues (typically the second and the last positions for HLA class I ligands) forming key interactions with the peptide-binding groove of HLA. In this study, we aimed to characterize HLA binding preferences in terms of molecular functions of presented proteins. To focus on the ligand presentation bias introduced specifically by HLA-peptide interaction we performed large-scale in silico predictions of binding of all peptides from human proteome for a wide range of HLA variants and established which functions are characteristic for proteins that are more or less preferentially presented by different HLA variants using statistical calculations and gene ontology (GO) analysis. We demonstrated marked distinctions between HLA variants in molecular functions of preferentially presented proteins (e.g. some HLA variants preferentially present membrane and receptor proteins, while others – ribosomal and DNA-binding proteins) and reduced presentation of extracellular matrix and collagen proteins by the majority of HLA variants. To explain these observations we demonstrated that HLA preferentially presents proteins enriched in amino acids which are required as anchor residues for the particular HLA variant. Our observations can be extrapolated to explain the protective effect of certain HLA alleles in infectious diseases, and we hypothesize that they can also explain susceptibility to certain autoimmune diseases and cancers. We demonstrate that these differences lead to differential presentation of HIV, influenza virus, SARS-CoV-1 and SARS-CoV-2 proteins by various HLA alleles. Taking into consideration that HLA alleles are inherited in haplotypes, we hypothesized that haplotypes composed of a combination of HLA variants with different presentation preferences should be more advantageous as they allow presenting a larger repertoire of peptides and avoiding holes in immunopeptidome. Indeed, we demonstrated that HLA-A/HLA-B and HLA-A/HLA-C haplotypes which have a high frequency in the human population are comprised of HLA variants that are more distinct in terms of functions of preferentially presented proteins than the control pairs.

## Introduction

T cells detect pathogen-infected and abnormal (e.g. tumour) cells by monitoring cell-surface-displayed short peptides presented by the human leukocyte antigen (HLA) complex. HLA molecules are highly specific in terms of the peptide sequences they are able to present, and peptides not presented by HLAs remain invisible to the immune system (1). HLA class I (HLA-I) and HLA class II (HLA-II) molecules present peptides that are typically recognised as a complex by CD8 and CD4 T cells, respectively; HLA-I-peptide complexes are also engaged by activating and inhibitory receptors on innate lymphocyte subsets such as natural killer (NK) cells. The three classical HLA-I genes expressed in all nucleated cells in humans are HLA-A, HLA-B, and HLA-C. HLA-I molecules present peptides derived from intracellular proteins. The intracellular antigen presentation pathway involves cleavage of proteins in the cytosol by proteasomes, translocation to the endoplasmic reticulum (ER) lumen, trimming by ER-resident aminopeptidases, loading onto HLA and presentation at the cell surface. Each cell’s HLAs present multiple different peptides at varying peptide-HLA copy numbers per cell. HLA-II genes (HLA-DR, HLA-DP and HLA-DQ) are constitutively expressed in only a subset of cells specialized for antigen presentation, such as dendritic cells, B cells, and macrophages, but expression can also be induced in additional cell types, e.g. in response to cytokine stimulation. HLA-II molecules present peptides derived from extracellular proteins taken into cells *via* endocytosis and phagocytosis, and intracellular proteins that access the HLA-II processing pathway *via* autophagy.

HLA-I molecules typically bind peptides of 8-12 amino acids (aa) in length. The HLA-I peptide-binding cleft is closed at both N- and C-terminal ends, and optimal length preferences are often biased towards binding of 9-mer peptides; longer peptides frequently bulge out of the cleft to be accommodated (2). For most HLA-I alleles the most abundant peptide length is 9 aa, but fine length preferences differ between alleles - in particular, some bind almost exclusively 8- and 9-mers (e.g., HLA-B*51:01) while others have a relatively high frequency of ligands of length 12-13 aa (e.g. HLA-A*01:01) (3). By contrast, HLA-II molecules possess an ‘open’ peptide-binding cleft and can therefore accommodate longer peptides than HLA-I. They frequently present nested sets of peptides that have a common “core” with N- and C-terminal extensions of varying length (4).

High affinity ligands for a given HLA allele usually share a common amino acid motif with relatively strict preferences in anchor positions (for HLA-I usually the second (P2) and last (PΩ), for HLA-II - P1, P4, P6 and P9), which form specific interactions with residues of corresponding HLA binding pockets (4, 5) The HLA locus is the most polymorphic in the human genome with tens of thousands alleles described to date (6). HLA variants that differ in peptide-contacting residues differ in the repertoire of peptides they present. The diversity of HLA alleles in the population is an important evolutionary mechanism for defense against diverse pathogens, e.g. rapidly mutating viruses. Different HLA alleles are associated with the severity and outcomes of viral infections. For example, the HLA-C*15:02 allele is associated with protection against SARS-CoV-1 (7), and HLA-B57 is highly associated with efficient HIV-1 control and long-term non-progressive infection in the absence of antiretroviral therapy (8).

Large-scale *in vitro* binding assays and recent advances in mass spectrometry (MS) have enabled generation of large datasets of ligands for many HLA alleles (5). The largest database of HLA ligands, IEDB (9), contains ~750,000 peptidic epitopes presented by 830 MHC alleles (as of August 2020).

Experimental HLA ligandome data is used for the training of artificial neural networks for prediction of HLA ligands and T cell epitopes [reviewed in (10, 11)]. Different tools, such as NetMHCpan (12) and MHCflurry (13) allow HLA-I ligand prediction with high accuracy and allow predictions even for HLA variants with no experimental data available (14). For HLA-II predictions of peptide binding are complicated by substantial variation in length of presented peptides and currently available HLA-II binding predictors have limited accuracy.

Comparison of MS-eluted HLA ligands and decoys predicted as HLA binders that were not observed in MS data enables the development of antigen processing predictors (13). The combination of antigen processing and HLA binding predictors in the MHCflurry tool resulted in significantly higher performance compared to HLA binding prediction only (13). Experimental HLA ligandome data (15) is also useful for the investigation of properties of proteins that are more likely to give rise to HLA ligands. It was recently shown that helical regions are significantly enriched in the ligands, suggesting different proteolytic resistance depending on the secondary structure and size of the initial protein fragment (16).

Apart from that, protein length and expression level, rate of proteasomal degradation, mRNA translation efficacy, presence of proteolytic signals, and sites of ubiquitination also influence the presence of protein-derived peptides in HLA ligandome (17, 18).

Several studies have employed gene ontology (GO) analysis to characterize functions of proteins that frequently serve as HLA ligands sources (17–23). These studies found enrichment of mitochondrion, ribosome, and nucleosome cellular component terms (18) and DNA-, RNA- and protein-interaction molecular function terms (17) and relative depletion of membrane and extracellular matrix proteins (21). However, these results may reflect differences in the expression level of the corresponding genes, rather than enrichment of HLA ligands within them. Abelin et al. (24) demonstrated that after correction for expression, enrichment in HLA ligands is observed only for proteins associated with the late endosome, although in the absence of the correction proteins with other localizations were also enriched (ER, mitochondria, nucleus, secreted) or depleted (cell membrane, cytoplasm). It was recently shown that most foreign MHC-I-displayed peptides are immunogenic (25). Additionally, recent work by the Cerundolo lab suggests that mitochondria-localized proteins are more immunogenic than other human peptides (26), which has implications for cancer immunotherapy. However, the studies mentioned above were based on aggregated datasets containing ligands from many distinct HLA alleles, and corresponding analyses were not focused on exploring differences between alleles. The datasets in question were also relatively small, e.g. in the largest of them (17) only 59% of human genes gave rise to at least one HLA ligand, while as it was recently shown by Sarkizova et al. (3) all human proteins may serve as sources of HLA ligands.

In this study, we investigated HLA binding preferences in terms of functions of presenting genes. In order to remove gene expression biases and focus on HLA presentation only, we used HLA ligandomes predicted *in silico* by the commonly used tool NetMHCpan-4.0, and focused mostly on HLA-I alleles due to significantly lower accuracy prediction for HLA-II. We performed HLA binding predictions for all possible peptides of length 8-12 derived from the human proteome for a set of HLA alleles with different binding motifs.

For all protein-coding genes, enrichment in HLA ligands was computed, and GO enrichment analysis was performed for sets of genes depleted or enriched in HLA ligands. Our results demonstrate that HLA alleles have a tendency to present peptides derived from proteins with specific molecular functions. These propensities are different for HLAs with different binding motifs, but similar for alleles with similar anchor residue preferences, which is explained by HLA preferential presentation of proteins enriched in amino acids that are favourable anchor residues for that allele.

Using experimental data from the HLA ligand atlas (15), we observe substantial differences between HLA class I and class II alleles, with class I alleles tending to present intracellular proteins and class II - membrane transport proteins.

Differences in functions of proteins preferentially presented by different HLA variants may be important for antiviral immunity. We demonstrate that HIV-protective HLA-B*57:01 is more likely to present proteins from GO categories corresponding to viral genes as compared with non-protective HLA-B*08:01.

We also hypothesized that HLA presentation bias towards proteins with specific functions may be compensated for in haplotypes. We demonstrate that HLA-A/HLA-B and HLA-A/HLA-C allele pairs from frequent HLA haplotypes are significantly more different in their GO enrichment profiles of the presented proteins than random allele pairs.

## Methods

### Predicting HLA ligands

All possible 8-12 mers were cut from the human proteome (UP000005640 record from UniProt database was used as a reference) and supplied to the software NetMHCpan v 4.0 (12) in order to predict putative HLA ligands for a set of 93 HLA alleles (see **Supplementary Table S1**) covering 95% of individuals worldwide. For the sake of brevity, proof-of-concept analysis and visualization was performed for a representative set of 6 HLA alleles (HLA-A*02:01, HLA-A*11:01, HLA-B*07:02, HLA-B*27:05, HLA-C*02:02, HLA-C*15:02), all having different anchor residue preferences (**Supplementary Figure S1**).

For exploration of differences in presentation of viral genes we expanded this set to 12 alleles with the addition of HLA-A*01:01, HLA-A*03:01, HLA-B*08:01, HLA-B*57:01, HLA-C*07:02 and HLA-C*08:01. Viral HLA-binding peptides were predicted using NetMHCpan v 4.0 software in the same way as human-derived ligands.

For analysis of compensation of HLA presentation bias in haplotypes, ligandome predictions were also made for alleles corresponding to frequent haplotypes but not presented in the set of 93 alleles. In total, 133 HLA alleles were surveyed in the current study.

The NetMHCpan software was run using default parameters, and both strong and weak binders (Rank < 2) were used as the list of putative human-derived ligands for each allele. Complementary analysis was performed using the MHCflurry software (13) with default parameters. In order to confirm that results were not biased by using a specific HLA binding prediction algorithm we selected HLA ligands based on the “Affinity percentile” column from MHCflurry. We also used the “Presentation score” column to account for antigen processing biases.

### HLA ligand enrichment analysis

Human proteins were assayed based on the number of observed and expected ligands for each HLA allele as follows. We first counted the number of predicted ligands *Ni* of length *l* coming from protein *i*. The average number of presented ligands for each HLA allele was computed as <*ρ* = <*N_i_* >/<*L_i_*, where *L_i_* = *length of protein* – *l* is the corrected protein length and <·> denotes the average over the proteome. The probability of observing a given number of ligands from each gene and the odds are computed using Binomial distribution as *P*(*N_i_*) = *P_binom_* (*N_i_* |*ρ*, *L_i_*) and *log Odds* = *log* (*N_i_* |*ρL_i_*). These values were used to define sets of HLA ligand-enriched and -depleted proteins (HLEPs and HLDPs).

### Experimentally validated HLA ligands

HLA ligands for both class I and class II alleles were extracted from the HLA Ligand Atlas dataset (15) that lists peptides obtained from publicly available MS HLA elution experimental data.

### Mass spectrometry-based profiling of peptides presented on single HLA-I allele-expressing cell lines

HLA-I-deficient CD4-expressing 721.221 cells (originally obtained from Prof Masafumi Takaguchi, Kumamoto University, Japan) were stably transfected with HLA-A*02:01. Transfectants were expanded by growth in RPMI 1640 medium (Thermo Fisher) containing 10% fetal bovine serum (FBS), 2 mM L-glutamine, 100 U/mL penicillin, and 100 μg/mL streptomycin (R10), and 2 x 10^8^ cells harvested for HLA-I bound peptide profiling. Mass spectrometry-based immunopeptidome profiling of HLA-A*11:01-transfected CD4.221 cells was reported previously (27); the same methodology was employed here for immunopeptidome profiling of HLA-A*02:01-expressing CD4.221 cells.

Briefly, cells were washed once in PBS, pelleted and 1 ml IGEPAL buffer [0.5% IGEPAL 630, 50 mM Tris pH8.0, 150 mM NaCl and 1 tablet Complete Protease Inhibitor Cocktail EDTA-free (Roche) per 10 ml buffer] was added per 0.5–1 × 10^8^ cells, and cells were lysed by mixing for 45 min at 4°C. Cell lysates were cleared by two centrifugation steps, 2000 × g for 10 min followed by 20,000 × g for 30 min at 4°C. HLA-peptide complexes were immunoprecipitated from the cell lysates on W6/32-coated Protein A Sepharose beads overnight at 4°C. W6/32-bound HLA-peptide complexes were sequentially washed with 20 mL of wash buffer 1 (0.005% IGEPAL, 50 mM Tris pH 8.0, 150 mM NaCl, 5 mM EDTA), wash buffer 2 (50 mM Tris pH 8.0, 150 mM NaCl), wash buffer 3 (50 mM Tris pH 8.0, 400 mM NaCl) and finally wash buffer 4 (50 mM Tris pH 8.0).

Peptide-HLA complexes were eluted from the beads in 5 mL of 10% acetic acid, and samples were dried down prior to resuspension in 120 μL loading buffer (0.1% TFA, 1% acetonitrile in ultragrade HPLC water). Samples were loaded onto a 4.6 × 50 mm ProSwift RP-1S column (Thermo Fisher Scientific) and eluted using a 500 μL/min flow rate over 10 min from 2 to 34% buffer B (0.1% TFA in acetonitrile) in buffer A (0.1% TFA in water) using an Ultimate 3000 HPLC system (Thermo Scientific). Alternate odd and even HPLC fractions were pooled and dried down prior to resuspension in 20 μL LC-MS/MS loading buffer (0.1% TFA in water).

For LC-MS/MS analysis, 9 ul of each sample was injected onto a Dionex Nano-Trap precolumn (Thermo Scientific), before separation with a 60 min linear gradient of acetonitrile in water of 2-25% across a 75 µm × 50 cm PepMap RSLC C18 EasySpray column (Thermo Scientific) at 40°C and a flow rate of 250 nl/min, resulting in an approximate average pressure of 600 bar. LC solvents contained 1%(v/v) DMSO and 0.1%(v/v) formic acid. Peptides were introduced using an Easy-Spray source at 2000V to a Fusion Lumos mass spectrometer (Thermo Scientific). The ion transfer tube temperature was set to 305°C. Full MS spectra were recorded from 300-1500 m/z in the Orbitrap at 120,000 resolution with an AGC target of 400,000. Precursor selection was performed using TopSpeed mode at a cycle time of 2 s. Peptide ions with a positive charge between 1-4 were isolated using an isolation width of 1.2 amu and trapped at a maximal injection time of 120 ms with an AGC target of 300,000. Singly charged ions were deprioritised to other ion species during acquisition. Higher-energy collisional dissociation (HCD) fragmentation was induced and fragments were analyzed in the Orbitrap. LC-MS/MS data was analysed using PEAKS v8.0 (Bioinformatic Solutions) software.

### Gene ontology enrichment analysis

Sets of proteins enriched and depleted in HLA ligands (HLEPs and HLDPs) were assayed for over-representation of certain Gene Ontology (GO) categories as follows. GO enrichment test was performed using the GOANA method from Limma R package (28) and top enriched GO terms coming from molecular function (MF), biological process (BP) and cellular component (CC) were selected for visualization. Additional verification of GO enrichment trends was performed with DAVID web tool (29). Note that while sets of HLEPs and HLDPs were used for GO analysis for *in silico* predicted ligands datasets, sets of proteins containing at least one HLA ligand were assayed for experimental data as most of those datasets contain too few ligands to perform ligand enrichment test.

### Analysis of compensation for HLA presentation bias in haplotypes

Data for HLA-A/HLA-B/HLA-C haplotypes with the highest frequency in 19 populations of different ethnic origin (listed in **Supplementary Table S2**, all from USA National Marrow Donor Program) was taken from the “Allele frequencies” database (http://www.allelefrequencies.net/) (30). Filtering for haplotypes with a frequency higher than 0.01% resulted in multiple entries for each of the populations (mean 42, range 28 - 89) which were merged to an aggregated dataset of 806 HLA-A/HLA-B/HLA-C haplotypes.

Further, these haplotypes were split into pairs of corresponding HLA-A/HLA-B, HLA-A/HLA-C, and HLA-B/HLA-C alleles (the resulting dataset is referred to as “Haplotypes”). The “Control” dataset included all possible HLA-A/HLA-B, HLA-A/HLA-C, and HLA-B/HLA-C combinations of unique alleles from the “Haplotypes” dataset with the exclusion of those pairs that were identical to real pairs from the dataset up to first two digits in allele names.

For each pair of alleles, the euclidean distance between GO term enrichment profiles was calculated and the distributions of that distance for “Haplotypes” and “Control” datasets were compared. GO term enrichment profile is a vector composed of enrichment folds for each of the analyzed GO terms (wherein fold value was taken with a positive sign for enriched GO terms, for depleted terms with a negative sign, and for not significantly changed terms fold was set to 0).

## Results

### Exploring differences in HLA ligand incidence across human proteins

**Figure 1:**
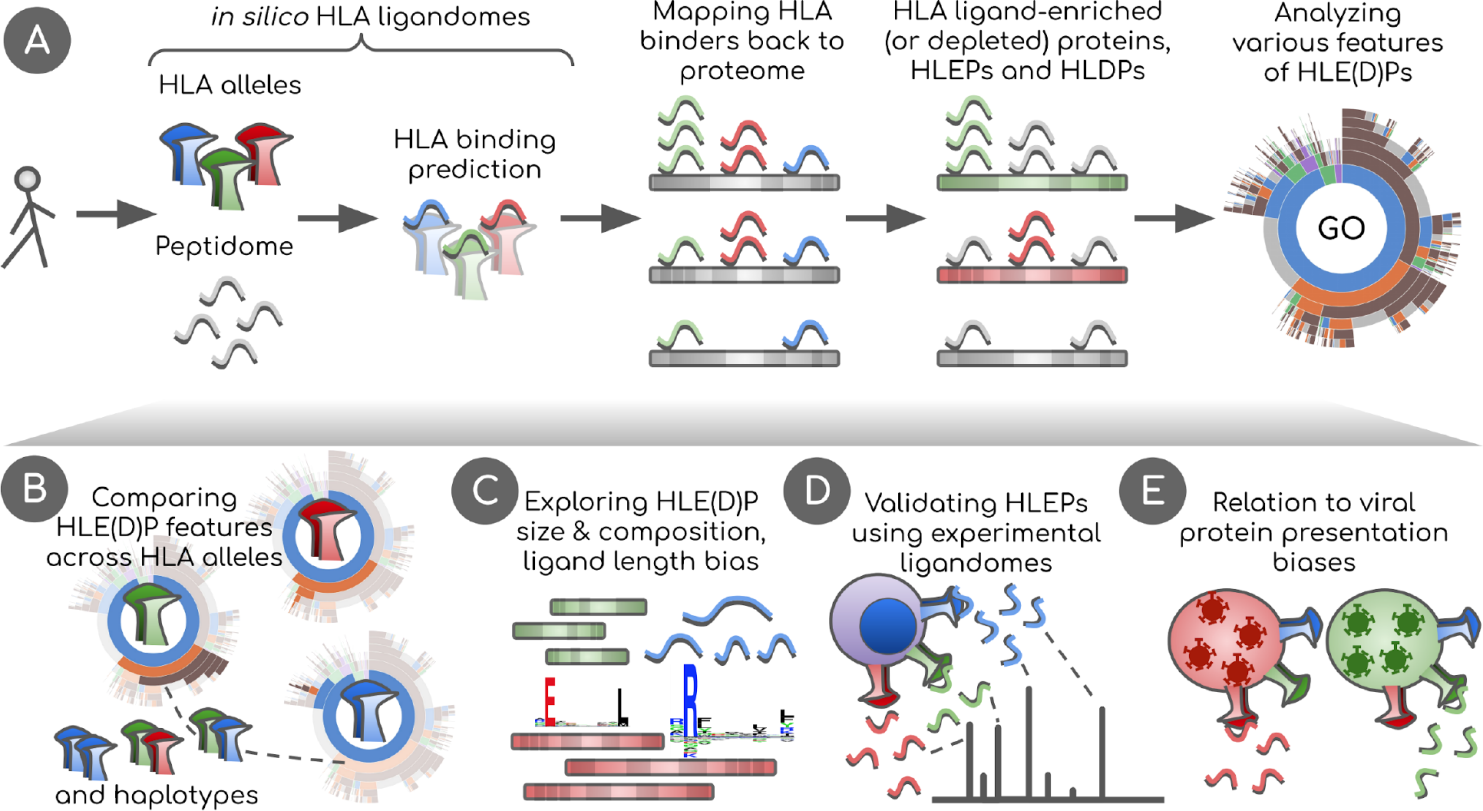
Overview of the study. **(A)** *In silico* HLA ligandomes are generated by running HLA binding prediction software for the human peptidome (8- to 12-mers). HLA binders are then mapped back onto their parent proteins. Statistical analysis is performed to define sets of human proteins enriched or depleted in HLA ligands, HLEPs and HLDPs respectively. Functional analysis of these gene sets is performed using Gene Ontology (GO) category enrichment tests. **(B)** GO annotation results are used to perform comparative analyses of HLA alleles, defining characteristic features of HLE(D)Ps. We show that preferred GO categories are clearly distinct between HLA alleles defining groups alleles with specific GO annotation profiles. These differences are however balanced and compensated by non-random selection of HLAs in HLA haplotypes observed in the population. **(C)** Potential biases that shape HLE(D)P sets are explored, such as protein length, protein amino acid composition, together with the length of HLA ligands and HLA anchor residue types. **(D)** Results are validated using real HLA ligandomes obtained from mass spectrometry data. **(E)** Differences are identified in non-self peptide presentation by various HLAs by studying HLA presentation preferences of viral proteins. We link viral and human peptide presentation by demonstrating the relation between self- and non-self protein presentation preferences for various HLAs.

We started our study (see **Figure 1** for overview) by running a large-scale *in silico* prediction of 9-mer HLA ligands in the entire human proteome using NetMHCpan software. We selected 93 HLA-A, HLA-B, and HLA-C alleles for our analysis (**Supplementary Table S1**) which cumulatively cover 95% of individuals worldwide based on allele frequencies reported by Sarkizova et al. (3). Our predictions yielded ~5x10^5^ HLA ligands for each allele (**Supplementary Table S3**) in line with previous estimates of the number of 9-mers a single HLA can present (31).

**Figure 2:**
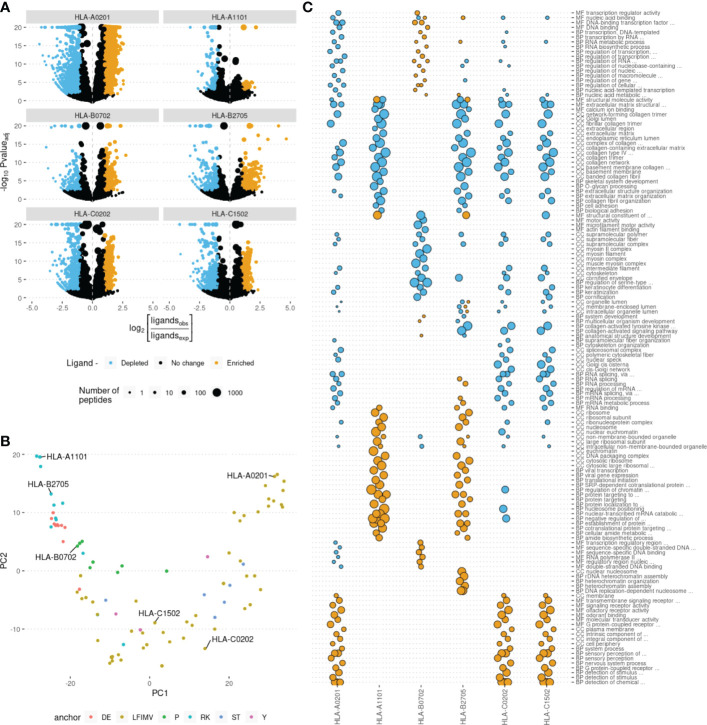
Human genes enriched and depleted in HLA ligands and their associated Gene Ontology (GO) categories. **(A)** Volcano plots showing the log of the ratio of observed to expected number of HLA ligands for each human gene plotted against enrichment P-value computed using binomial test. Point size shows the number of predicted HLA ligands, point color highlights genes enriched and depleted in ligands according to at least 2-fold increase or decrease in the number of ligands and adjusted P-value of < 0.05. Data for 6 selected HLA alleles are shown as separate plots. **(B)** Principal component analysis (PCA) of gene ontology (GO) term enrichment profiles. Each point represents one of 93 common HLA alleles. Colors correspond to physico-chemical properties of required anchor residues. 6 representative alleles, selected for further analysis, are marked. **(C)** GO term enrichment analysis for human genes differentially presented by different HLAs. Point size represents the GO enrichment fold for genes enriched (yellow) and depleted (blue) in HLA ligands for each of 6 surveyed HLA alleles. An adjusted P-value threshold of 0.01 was used as a threshold, Y axis lists the union of sets of top 20 GO categories for both ligand-enriched and ligand-depleted genes for each HLA allele. GO term names are preceded by either CC (cellular component), MF (molecular function) or BP (biological process) ontology name.

For each of ~20,000 human proteins we calculated the number of peptides derived from them that were predicted to be binders to each of HLA alleles. We noticed that some proteins yield more or less HLA ligands than expected by chance under the assumption of uniform coverage of the proteome with HLA ligands. We termed such proteins as HLA ligand-enriched proteins (HLEPs) and HLA ligand-depleted proteins (HLDPs). To statistically define HLEPs and HLDPs for each HLA allele we computed the number of ligands mapping to every human protein and estimated the expected number of ligands for each protein as the proteome-average ligands-per-amino acid frequency multiplied by the length of the protein. HLEPs and HLDPs were then selected based on a fixed P-value threshold (computed using Binomial distribution and adjusted for multiple testing, see **Methods** section) and the observed-to-expected ratio of mapped ligand counts (**Figure 2A**).

We next analyzed the length distribution HLEPs, HLDPs and the remaining proteins that do not show any difference in HLA ligand counts (**Supplementary Figure S2**). While one might expect that longer proteins would provide more statistical power to infer differences in the number of HLA ligands, we found that proteins of any length can feature differences in HLA presentation. More specifically, we observed that longer proteins are more likely to be depleted in ligands while shorter ones are enriched in presented peptides.

### HLAs differentially present human proteins associated with certain gene ontology categories

Further we hypothesized that HLEPs and HLDPs may be linked to subsets of human proteins characterized with some common molecular functions. In order to explore these preferences we ran GO term enrichment analysis for sets of HLEPs and HLDPs for each HLA allele. We summarized the results of this analysis as a matrix containing GO enrichment folds as entries for each of GO terms (columns) and each of 93 surveyed HLA alleles (rows). We visualized similarity between alleles using principal component analysis (PCA) of matrix entries (**Figure 2B**). Thus, alleles which preferentially present proteins with similar functions are located nearby on this PCA plot. We also noticed that the alleles are clustered according to physico-chemical properties of required anchor residues. Interestingly, not all of the similarities can be fully explained by shared ligandomes: for example, HLA-A*11:01 and HLA-B*27:05 have distinct peptide-binding motifs (with K9 anchor and R2 anchor, respectively; see **Supplementary Figure S1**) and almost no common ligands, however, they have similar preferences in terms of functions of presented proteins.

We also performed an in-depth analysis for representative set of 6 HLA alleles which are located at distinct regions of PCA plot in **Figure 2B**: two HLA-A alleles (HLA-A*02:01 and HLA-A*11:01), two HLA-B alleles (HLA-B*07:02 and HLA-B*27:05) and two HLA-C alleles (HLA-C*02:02 and HLA-C*15:02).

For 6 representative alleles we detected on average 877 HLEPs (range from 55 to 1946) and 565 HLDPs (from 106 to 1557, see **Supplementary Table S3**). We observed prominent enrichment of certain “molecular function” (MF), “biological process” (BP) and “cellular component” (CC) GO categories of genes coding for HLEPs and HLDPs and found differences between GO category profiles across surveyed HLAs.

In general, genes coding for HLDPs are more likely to encode extracellular matrix components, collagen and myosin, which is in line with the observation mentioned above as those are typically longer proteins. It is thus hard to decouple potential length bias from gene function in this case, as all HLA alleles show similar disfavoring of this set of genes.

On the other hand, genes coding for HLEPs display a diverse set of associated GO categories. For example, HLA-A*02:01, HLA-C*02:02 and HLA-C*15:02 are more likely to present ligands from genes encoding membrane proteins and those involved in receptor signalling such as G-coupled receptor and olfactory receptor signalling. HLEPs for HLA-A*11:01 and HLA-B*27:05 are involved in translation and gene expression, while HLA-B*07:02 ligands derive from proteins involved in regulation of transcription and HLA-B*27:05 presents ligands from genes involved in DNA replication and chromatin silencing. It is also necessary to note that HLA-A, -B and -C genes do not show much similarity and alleles of different HLA-I genes can have similar preferences.

### Human proteins are differentially represented within HLA ligands of different length

In order to check for differences in human HLEP and HLDP set composition for HLA ligandomes corresponding to different peptide lengths we surveyed 8-mer to 12-mer predictions for the HLA-A*11:01 allele as described above (**Supplementary Figure S3**). HLA-A*11:01 predominantly presents 9- and 10-mers, although longer and shorter peptides are also known to be presented by this allele (32). We found genes that were either enriched or depleted for HLA ligands for all surveyed peptide lengths; the total number of ligands of each length was around 10^5^ (**Supplementary Table S4**). Analysis of genes enriched within HLA-A*11:01 ligands of each length reveals a number of GO categories that are associated with longer and shorter HLA ligands (**Supplementary Figure S4**). GO categories characteristic of genes depleted in HLA-A*11:01 ligands are similar across all ligand lengths and correspond to genes coding for extracellular proteins and collagen, in line with general trends observed for the 6 HLA-I alleles described above. GO categories of genes coding for HLA-A*11:01 HLEPs are, however, distinct across peptide lengths: 9- and 10-mer peptides are linked to genes involved in transcription and translation processes, while 12-mers are linked to genes associated with mitochondrial and transporter genes. As observed biases in HLEP features may be due to differences in *in silico* ligand prediction accuracy for different lengths (for example, there are far more training examples of 9-mer ligands than 11-mers (12)), we performed additional validation of these results using experimental HLA ligandomes and alternative software tools as described in the next sections.

### Amino acid composition of proteins enriched or depleted in HLA ligands

**Figure 3:**
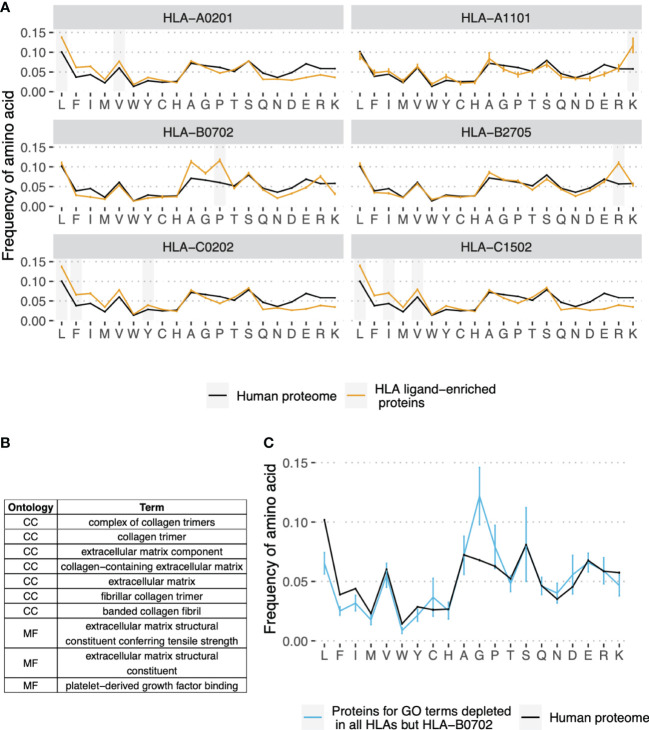
Amino acid composition of HLEPs and HLDPs for different HLA alleles. **(A)** Comparison of amino acid composition of HLEPs for the 6 selected HLA alleles and all proteins of the human proteome. Grey bars mark amino acids preferred by the allele in the anchor positions (P2 and/or P9) according to **Supplementary Figure 1**. Note that HLEPs tend to have a higher frequency of amino acids that are good anchors for this allele. Error bars show 95% confidence interval for the mean value. **(B)** GO categories associated with HLDPs for 5 of 6 selected alleles (all but HLA-B*07:02). Ontology names: CC - cellular component, MF - molecular function, BP - biological process. **(C)** Comparison of amino acid composition of proteins corresponding to GO categories from **(B)** (only proteins which are in HLDPs for at least 1 allele were considered) and human proteome. Error bars show 95% confidence intervals for the mean value. Amino acids which are enriched in proteins corresponding to “commonly depleted” GO categories are glycine which can’t be used as anchors for most HLA alleles, and proline which dramatically affect peptide backbone conformation. HLA-B*07:02 represents a special case as this allele strictly prefers proline as anchor residue in P2.

To explore the molecular basis for differences in gene presentation profiles between HLA alleles, we compared the amino acid composition of HLEPs and HLDPs. The results presented in **Figure 3A** demonstrate that HLEPs are enriched in amino acids which are good anchor residues for the particular HLA allele (see **Supplementary Figure S1** for motifs of presented peptides). For example, for HLA-A*02:01, HLA-C*02:02, and HLA-C*15:02, which require hydrophobic anchors, HLEPs have a higher frequency of hydrophobic and lower frequency of charged residues. The amino acid frequency profile for HLA-B*27:05 HLEPs is very close to that of the human proteome except for a higher frequency of arginine, which is strictly preferred by the allele as an anchor residue in the P2 position.

The observed bias in the amino acid composition of HLEPs and HLDPs leads to differences in the GO categories enriched in these gene sets. Thus, for HLA-A*02:01, HLA-C*02:02 and HLA-C*15:02, which are prone to present more hydrophobic proteins, GO categories enriched in HLEPs are mainly associated with membrane proteins (**Figure 2B**) which have a relatively high frequency of hydrophobic residues. HLA-A*11:01 and HLA-B*27:05, which require lysine and arginine as anchor residues, are more likely to present proteins involved in interaction with DNA (**Figure 2B**) and that have a relatively high frequency of positively charged amino acids.

Comparing GO enrichment analysis results for different alleles we found that several GO categories are enriched in HLDPs for all surveyed alleles except HLA-B*07:02 (**Figures 2B** and **3B**). Genes corresponding to these GO terms are enriched in glycine and proline residues as shown in **Figure 3C**. These GO categories are mostly associated with the extracellular matrix (**Figures 2B** and **3B**) and include fibrous proteins such as collagen. Glycine and proline residues are critically important for collagen ternary structure formation. Glycine is a bad anchor residue for almost all HLAs, and proline dramatically affects peptide conformation preventing its binding with HLA. It can be suggested that proteins enriched in G and/or P are poorly presented by multiple HLAs. HLA-B*07:02 is the exception as this allele strictly requires proline as the peptide P2 anchor.

To better understand the protein length bias for HLEPs and HLDPs we compared the amino acid composition of human proteins of different lengths. As shown in **Supplementary Figure S6A**, the frequency of hydrophobic amino acids is highest for proteins in the second length quartile (Q2) and lowest for Q4 proteins. In accordance with this trend Q2 proteins constitute the highest fraction of HLEPs for HLA-A*02:01, HLA-C*02:02, and HLA-C*15:02 alleles featuring hydrophobic anchor residues. For HLA-A*11:01 and HLA-B*27:05, which utilise positively charged anchor residues, Q1 proteins constitute more than half of HLEPs, in line with the observation that these proteins have the highest frequency of arginines and lysines.

### Exploration of ligand presentation bias on an extended dataset of HLA alleles

To check that the conclusions from the analyses in the previous sections are not artifacts of the selection of surveyed HLA alleles, we performed *in silico* predictions of HLA ligands and GO enrichment analysis for an extended set of 93 alleles (**Supplementary Table S1**).

First, we re-examined our observation of the existence of GO categories associated with HLDPs for most alleles. We observed that there is a group of GO terms related to extracellular matrix and collagen which are associated with HLDPs for up to 95% (89 out of 93) of surveyed alleles (**Supplementary Figure S5A**). Proteins corresponding to these terms are enriched in glycines and prolines (**Supplementary Figure S5C**). Exceptional alleles, for which these “universally depleted” terms are not associated with HLDPs are the ones that strictly require proline residues as P2 anchors (**Supplementary Figure S5B**). Overall, all the conclusions reached from the analyses performed with the initial set of 6 alleles, i.e. depletion of proteins enriched in glycines and prolines which correspond to GO terms related to structural functions (**Figures 3B, C**, with the exception of HLA-B*07:02 having proline anchor residues), remain valid when a broader range of alleles are considered.

Further, we explored HLA presentation preferences for proteins of different lengths. For the majority of alleles, the highest fraction of HLEPs is composed of smaller proteins from the second length quartile (Q2) and most HLDPs are longer proteins from Q4 (**Supplementary Figure S6B**). Preferences in the length of presented proteins may be explained by the differential amino acid composition of proteins from different length quartiles (**Supplementary Figure S6A**). Hydrophobic amino acids which are required as anchor residues for the majority of alleles (see **Supplementary Table S1**) are enriched in Q2, so for these alleles the length distribution of HLEPs peaks in Q2. There are some exceptions where the distribution of length of HLEPs is not maximal in Q2 (**Supplementary Figure S6C**), but these exceptions are also explained by the amino acid composition of proteins in different length quartiles. Alleles for which length distribution of HLEPs peaks in Q1 require positively charged anchor residues (arginine and lysine) which are enriched in Q1 proteins. Alleles that prefer to present Q4 proteins require negatively charged glutamic acid as an anchor which is enriched in Q4 proteins (**Supplementary Figure S6A**).

### Validation of HLA allele and ligand length biases

To ensure our observations are not an artifact of the HLA ligand prediction model used by NetMHCpan software, we re-ran the same analysis using an alternative software tool MHCflurry (13). In addition to peptide-HLA binding, MHCflurry also takes into account antigen processing and can provide the combined score (“Presentation score”), which performs better to predict HLA ligands. We performed the analysis using both affinity and presentation scores of MHCflurry to enable evaluation of the impact of software and potential antigen processing biases. In both cases the analysis revealed nearly identical results to **Figure 2** in terms of ligand enrichment scores and P-values, as well as the list of associated GO categories, as can be seen in **Supplementary Note 1, Figure SN1**. Also, the bias in the number of HLA ligands reported for proteins of different lengths (**Supplementary Figure S2**) holds true when applying MHCflurry as a HLA ligand prediction method (**Supplementary Note 1, Figure SN2**).

In addition, to determine whether the interpretation of our findings would be affected by use of an alternative GO annotation strategy, we re-annotated HLA ligand-enriched and -depleted gene sets using DAVID, another commonly used web tool (33). DAVID analysis showed clustering of GO categories enriched and depleted for genes of interest that was highly in line with the results described above (**Supplementary Note 1, Figure SN3**). Annotation clusters (groups of GO categories) identified by DAVID as being over-represented are also supportive of the general trends mentioned above, e.g. depletion of proteins representing extracellular matrix components, collagen and myosin in HLA ligands.

**Figure 4:**
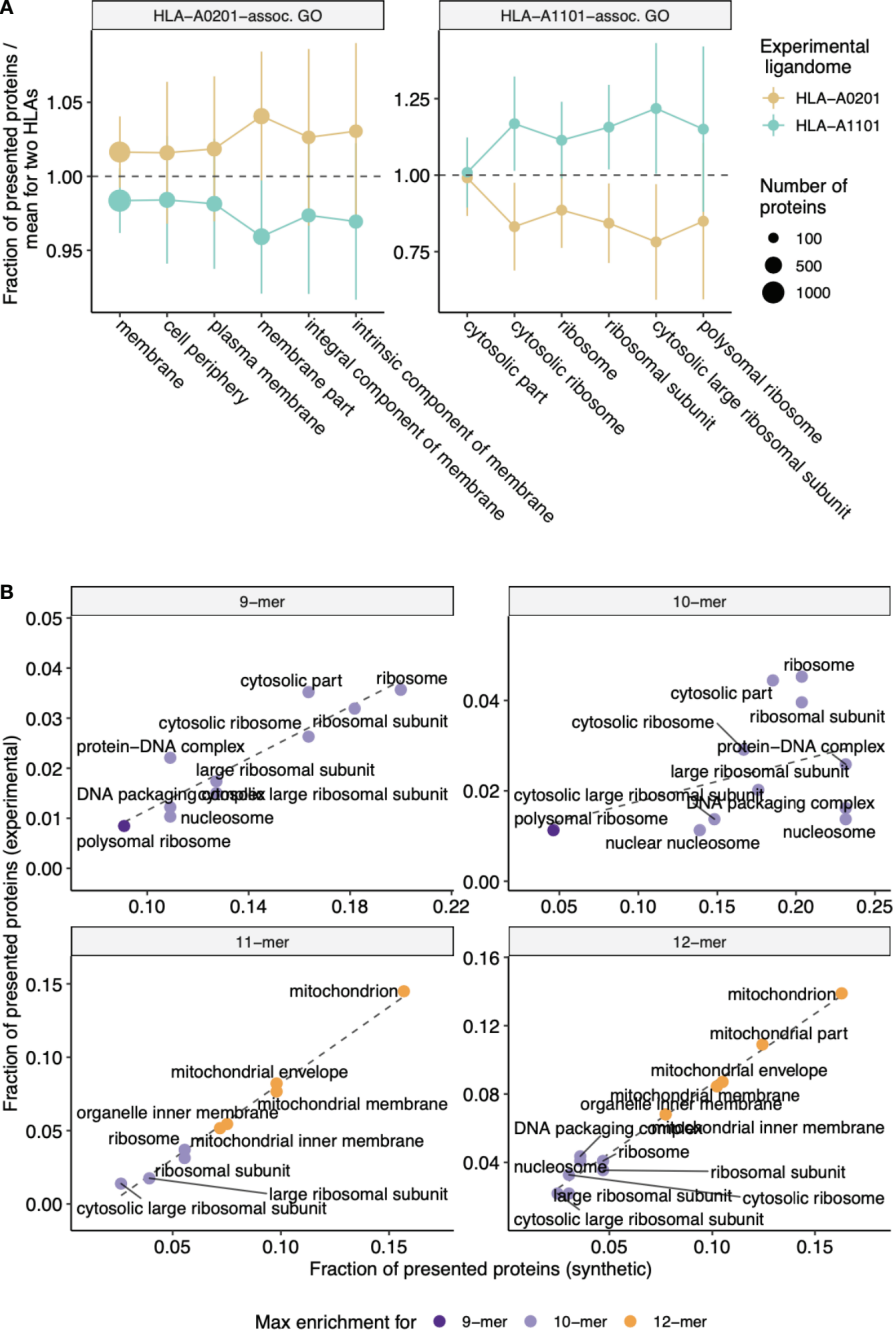
Experimental validation of biased selection of self proteins presented by different HLA alleles and different HLA ligand lengths. **(A)** Fraction of proteins presented by experimentally obtained HLA-A*02:01 and HLA-A*11:01 ligandomes that correspond to a given GO category. The fraction is normalized by mean value for two alleles to highlight differences between proteins related to HLA-A*02:01 and HLA-A*11:01 alleles. Cellular component (CC) GO categories associated with proteins which are frequently presented by either HLA-A*02:01 (left panel) or HLA-A*11:01 (right panel) according to *in silico* predictions were selected to match those in **Figure 2B**. Error bars show 1 standard deviation of fractions. **(B)** Scatterplot compares the fraction of proteins presented by HLA-A*11:01 ligands of various lengths that correspond to a given GO category between *in silico* predictions and experimental data. CC GO categories associated with 9-12-mer ligands were selected to match those in **Supplementary Figure 5**. Color shows ligand length at which maximum fold enrichment is reached for a given GO category.

We also independently validated our results with experimentally-determined ligandomes of HLA-A*02:01 and HLA-A*11:01 alleles (see **Methods** section). Analysis of GO categories enriched in parent proteins of HLA-A*02:01 peptides compared to HLA-A*11:01-presented proteins and vice-versa revealed consistency with the *in silico* results reported above, and protein GO categories matched HLEPs of corresponding HLA alleles. As can be seen from **Figure 4A**, GO categories that are enriched in either HLA-A*02:01 or HLA-A*11:01 according to *in silico* data analysis are also more common in proteins that feature HLA ligands of corresponding alleles in experimental data, supporting the observed difference between functions of HLA-A*02:01 and HLA-A*11:01 HLEPs. Moreover, GO categories enriched in HLEPs of HLA-A*11:01 allele ligands of various lengths are highly correlated with GO categories enriched for ligands of corresponding lengths in experimental data (**Figure 4B**).

### HLA ligand atlas analysis and difference between class I and class II HLAs

To provide further verification of our results on real HLA ligand datasets and compare them to *in silico* HLA ligand predictions we explored the HLA ligand atlas dataset (15). We ran GO enrichment analysis for sets of genes corresponding to peptides presented by each HLA allele in the dataset and compared profiles of enriched categories across alleles. Note that we used every gene that has at least one reported HLA ligand, and did not use an enrichment test for the number of ligands per gene as the size of the database is too small to ensure good coverage of all human genes. Using HLA ligand atlas allowed us to independently validate the phenomenon of preferential presentation of genes with specific functions by different HLA alleles.

**Figure 5:**
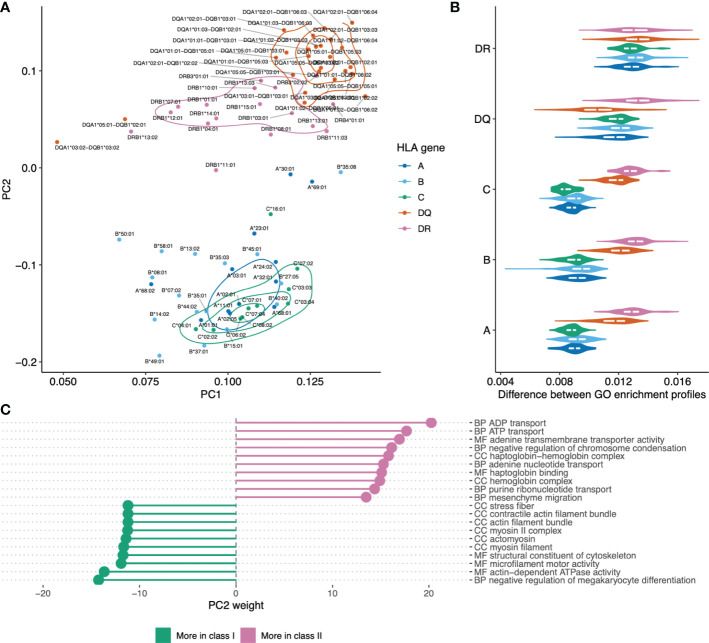
Visualizing similarities between HLA alleles based on enriched GO categories of genes they tend to present. **(A)** Principal component analysis (PCA) results for GO category fold enrichment profiles of various HLAs. GO enrichment profiles are computed based on gene sets obtained by mapping HLA ligands from the HLA ligand atlas dataset as the logarithm of observed to expected fraction of genes representing a given GO category. Color shows HLA gene: A/B/C for class I and DR/DQ for class II. **(B)** Distribution of pairwise distances between GO enrichment profiles of HLA alleles of the same and distinct HLA genes. Y axis corresponds to the HLA gene of the first HLA allele in each pair, the gene of the second allele indicated by color (same as in **A**). Euclidean distance divided by the total number of GO categories is used. **(С)** List of the top 10 GO categories that have most absolute weight in PC2 (see panel **A**). Negative weight corresponds to dominance in class I HLA alleles, while positive weight corresponds to dominance in HLA class II. GO term names are preceded by either CC (cellular component), MF (molecular function) or BP (biological process) ontology name.

Principal component analysis was used to visualize differences between HLAs based on functions of genes they tend to present as shown in **Figure 5A**. The plot shows clear separation between genes providing a source of ligands presented by HLA class I and class II, with co-clustering of HLA-C and HLA-B alleles and a notable differentiation between HLA-DQ versus HLA-DR. Notably, while HLA class I and class II alleles are clearly separable, it is hard to tell HLA class I genes apart based on GO enrichment profiles (**Figure 5B**).

For in-depth exploration of genes that are differentially presented between class I and class II alleles we took the PC2 component from **Figure 5A** that linearly separates HLA classes and analyzed GO categories having highest weights in these components (**Figure 5С**). We observed that class II HLA alleles are more likely to present membrane transport proteins, while class I alleles are prone to present components of intracellular structural proteins.

### Analyzing presentation of viral genes by different HLA alleles

**Figure 6:**
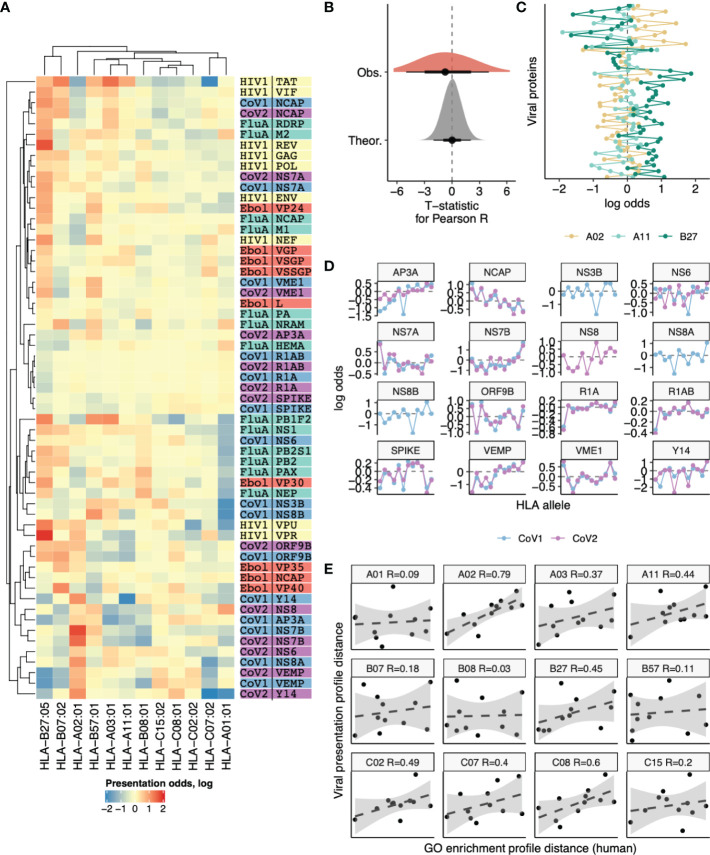
Differences in the number of ligands coming from viral genes presented by 12 different HLA alleles. **(A)** Heatmap showing the logarithm of the ratio of observed to expected number of HLA ligands (presentation odds). Expected number of ligands for each gene and HLA pair was estimated as the sum of corresponding row and column of the matrix divided by the total number of ligands in the matrix. Dendrograms show results of hierarchical clustering of gene-wise and HLA-wise presentation profiles performed using complete linkage algorithm with Euclidean distance measure. Proteins from HIV (yellow), Influenza (green), Ebola (red), SARS-CoV-1 (blue) and SARS-CoV-2 (purple) are shown. **(B)** Absolute values of T-statistic for observed pairwise correlation coefficients between viral gene presentation profiles of different HLAs (dark grey) compared to theoretical distribution (n=1000 random samples from null distribution with same number of degrees of freedom, light grey). **(C)** An example comparison of viral gene presentation profiles between HLA-A*02:01, HLA-A*11:01 and HLA-B*27:05 alleles. **(D)** Comparison of SARS-CoV-1 and SARS-CoV-2 protein presentation odds across different HLA alleles. **(E)** Correlation between distance in viral protein presentation odds profiles (Y axis) and distance in GO category profiles of HLA ligand-enriched human genes (X axis) for all HLA alleles. Each panel shows distances from a given HLA profile to profiles of each of the remaining 11 HLA alleles. Allele name and Pearson correlation coefficient are shown in panel title.

As the spectrum of viral protein functions should be highly specific we suggest that our observation of differential presentation of peptides derived from human proteins with differing functions may be extrapolated to presentation of viral peptides by distinct HLAs. Thus, we suggested that there may be substantial differences in presentation of viral peptides and different human HLA alleles will favor certain viral proteins. We calculated the presentation odds for each viral gene-HLA pair as the ratio of the observed number of ligands divided by expected value computed under the assumption of independence between an HLA allele and the number of ligands it presents from a given gene. Comparative analysis of presentation odds of viral peptides by human HLAs (**Figure 6A**) reveals co-clustering of viral genes with similar functions and certain HLAs (e.g. HLA-B*07:02 and HLA-B*27:05, HLA-A*03:01 and HLA-A*11:01).

However, surveyed HLA alleles mostly feature contrasting presentation odds profiles, and the distribution of correlation coefficients for these profiles is shifted to negative values (**Figure 6B**). For example a pair of HLA-A alleles, HLA-A*02:01 and HLA-A*11:01, appear to have distinct preferences for presenting viral proteins (**Figure 6C**), in line with their difference in preferences for presenting human proteins with certain functions reported above (**Figure 2B**). On the other hand, HLA-A*11:01 and HLA-B*27:05, which tend to present human proteins of similar functions (**Figure 2B**) are also similar in terms of viral protein presentation odds profiles (**Figure 6C**).

Considering similar viral proteins, we observe little difference in the way they are presented by the same HLA. When comparing presentation odds across all 12 surveyed HLAs between proteins of SARS-CoV-1 and SARS-CoV-2 strains we observe nearly perfect correlation for almost all proteins (**Figure 6D**).

Finally, we performed a direct comparison to test the hypothesis that tendency to present self-peptides with certain functions is intrinsically linked to the variability in viral protein presentation by HLAs. We tested if distance between self-peptide GO profiles was correlated with distance in viral presentation profiles, testing each of 12 HLA alleles against the remaining 11 (**Figure 6E**). All alleles show a positive correlation between these two distances, and for the majority the correlation was substantial (R > 0.3 for 7 out of 12 alleles). The overall correlation coefficient for all 66 possible distance pairs is R = 0.32, P = 0.009.

We can speculate that inter-allele differences in preferences for binding of peptides derived from viral as well as human proteins could be among the factors contributing to the differential association of particular alleles with protection/pathogenesis in different infections (see **Discussion**)

### Haplotype compensation of bias in HLA presentation of proteins with different molecular functions

In previous sections, it was noted that HLA alleles with similar anchor residue preferences have similar profiles of GO terms enrichment and viral gene presentation odds, while alleles with different anchor preferences are more likely to have contrasting profiles (see **Figures 2B** and **6A-C**). Considering that HLA alleles are inherited not individually but in haplotypes, one may hypothesize that haplotypes composed of HLA alleles that are prone to present proteins with different molecular functions might be evolutionarily advantageous as they would be able to present a more diverse set of peptides to the immune system.

**Figure 7:**
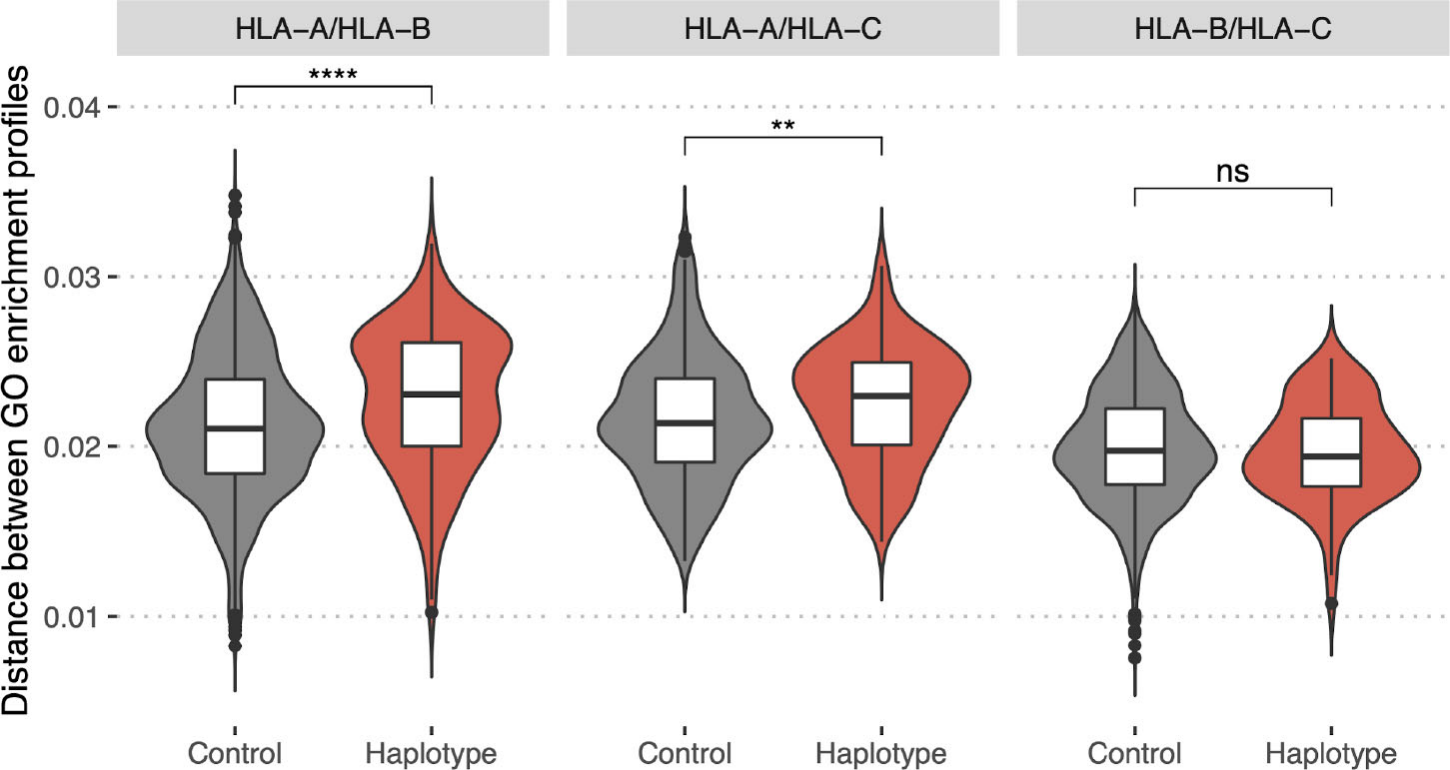
Bias in HLA presentation of proteins with different molecular functions is compensated in HLA-A/HLA-B and HLA-A/HLA-C haplotypes. Distance in GO category profiles between pairs of HLA alleles of different genes (HLA-A and HLA-B, left panel; HLA-A and HLA-C, central panel; HLA-B and HLA-C, right panel). “Haplotype” set is composed of pairs of alleles constituting haplotypes with high frequency in one of the populations, “Control” is composed of random pairs of alleles from the “Haplotype” set (for details see Methods section). Statistically significant differences between the groups are for HLA-A/HLA-B (p-value = 5e-07, Mann–Whitney U-test) and HLA-A/HLA-C genes (p-value = 0.004, Mann–Whitney U-test). Higher values of the distance for “Haplotype” group indicate that pairs of alleles composing frequent haplotypes tend to present proteins with distinct functions. (****P ≤ 0.0001; **P ≤ 0.01; ns, not significant).

To test this we collected a dataset of HLA class I haplotypes (combinations of HLA-A, HLA-B, and HLA-C alleles) which have the highest frequencies in populations of different ethnic origin (**Supplementary Table S2**, for details see **Methods** section). Haplotypes were divided into pairs of alleles of different genes (HLA-A/HLA-B, HLA-A/HLA-C, and HLA-B/HLA-C). As a control set we reshuffled alleles from the haplotype set to make up random pairs. In both sets, for each resulting pair of HLA alleles, we computed GO terms enrichment profiles distance. A comparison of corresponding distributions (**Figure 7**) between these two sets demonstrates that pairs of HLA-A/HLA-B and HLA-A/HLA-C alleles associated with frequent haplotypes are significantly different in terms of the distance between corresponding GO terms enrichment profiles from control allele pairs. Thus, commonly observed haplotypes consist of more divergent pairs of HLA alleles in terms of the proteins they tend to present peptides from. This result may be interpreted as indicating that the haplotype composition is focused on compensating “holes” in the immunopeptidome that are the result of non-uniform proteome presentation by various HLAs.

## Discussion

T cell recognition of antigenic peptides presented by HLA proteins is critical for protection against viruses and other pathogens, elimination of cancer cells and prevention of autoimmune diseases, moreover it directly shapes the T cell repertoire that form the backbone of adaptive immunity (1, 34–36). HLA locus is the most polymorphic in the human genome and different HLA variants present different sets of peptides. The human proteome is not uniformly represented within HLA ligands and some proteins may give rise to more or less HLA ligands than it is expected from random sampling. One might suppose that the choice of such preferentially presented or depleted proteins is not stochastic but can be characterized by some specific functions (which may be specified as gene ontology (GO) categories). Several studies have been aimed to characterize GO categories of the proteins which are preferentially presented by HLA (17–23). However, all of them were based on experimental datasets of MS-derived HLA ligandoms and thus are strongly influenced by protein expression bias. Also, in such datasets the number of detected ligands is too small for each individual HLA variant so it is not feasible to investigate the differences between different HLA alleles because of the lack of statistical power. To overcome this issue, we based our analysis on *in silico* predicted HLA ligandomes. We performed large-scale predictions of binding of all possible peptides derived from human proteome (~10^7^ peptides from ~2*10^4^ proteins) and 93 common HLA variants covering 95% of individuals worldwide, using NetMHCpan or MHCflurry software which have very high accuracy. We observed that a number of proteins are more or less preferentially presented by certain HLA alleles and that these sets of proteins can be characterized by specific molecular functions. Interestingly, HLA variants substantially differ in molecular functions of preferentially presented proteins. We explain these differences by the tendency of HLA to preferentially present proteins with a higher fraction of amino acids which are good anchor residues for that particular allele. For example, HLA variants which require hydrophobic anchors (e.g., HLA-A*02:01, HLA-C*02:02, and HLA-C*15:02) preferentially present hydrophobic proteins and in particular membrane, signaling and sensory proteins. HLA variants with positively charged anchors (e.g., HLA-A*11:01 and HLA-B*27:05) preferentially present proteins enriched in positively charged residues, such as those that bind negatively charged DNA and RNA. Of note, HLA-A*11:01 and HLA-B*27:05 have substantially different anchor residue preferences (with lysine in P9 and arginine in P2, respectively), but since for both alleles the anchors are positively charged, they have similar preferences in terms of functions of presented proteins. Interestingly, extracellular matrix, structural and collagen proteins were depleted in HLA ligands for the majority of HLA alleles. This is explained by the fact that these proteins have a relatively high proportion of glycine residues (which are disfavoured anchor residues for almost all HLA alleles) and prolines (which are conformationally rigid and may disturb peptide conformation suitable for HLA binding). Notably, for HLA variants which require proline anchor residues (e.g., HLA-B*07:02) these proteins are normally presented.

Also, our analysis demonstrated that proteins of shorter lengths are more preferentially presented by the majority of common HLA variants compared to the longer proteins. This result might have a biological interpretation: since viral and bacterial proteins are typically of shorter length compared to human proteins, such a length bias may facilitate effective immune defense from pathogens and avoid autoimmune reactions.

To account for potential bioinformatics software biases, we re-ran the analysis using alternative software for prediction of peptide-HLA binding and another tool for GO annotation as well as using experimental data and confirmed robustness of our results.

Different preferences in functions of presenting proteins between HLA alleles may be important for antiviral immunity. We speculate that differences in the tendency of HLA alleles to present peptides from proteins with certain functions may be among the factors contributing to differential association of alleles with infection outcomes. To illustrate this, we compared binding preferences of HLA-B*57:01, associated with a good disease prognosis in HIV-1 infection, and HLA-B*08:01, associated with a poor prognosis (8). This analysis showed that beneficial HLA-B*57:01 variant preferentially present proteins with membrane and ion transport-related functions that are assigned to HIV proteins (**Supplementary Figure S7**).

Intriguingly, we show that the reported HLA presentation bias is compensated for in frequent haplotypes. HLA-A, HLA-B and HLA-C genes are located close to each other on the same chromosome, so they are inherited not independently, but as a haplotype. Taking this into consideration, we hypothesized that haplotypes with a high frequency in population should be composed of HLA alleles with complementary peptide-binding preferences that would result in an increased size of the immunopeptidome presented in each individual. Indeed, we demonstrated that HLA-A/HLA-B and HLA-A/HLA-C pairs in frequent haplotypes are statistically more distinct in profiles of binding preferences compared to control (swapped) pairs. The absence of the effect for HLA-B/HLA-C pairs may be explained by the fact that different HLA-B and HLA-C alleles are more similar to each other than either of them are to HLA-A in terms of amino acid sequence, a result of the evolutionary origin of the HLA-C gene being from duplication of HLA-B (37). These results may suggest that haplotypes of HLA alleles with different preferences for presenting proteins with particular molecular functions are evolutionarily beneficial and have a greater chance of becoming fixed in the population. This result is in line with previous studies demonstrated that the mechanisms of divergent allele advantage (38) and heterozygous advantage (39) impacted the evolution of HLA and that patients with heigher sequence divergence of carried HLA alleles have better response to immunotherapy (40).

It should be noted that in a 2013 paper Rao et al. (41) reported that complementarity of binding motifs in frequent HLA-A/HLA-B haplotypes is not higher than in random HLA-A/HLA-B pairs. The difference between earlier results and our conclusions may be explained by the lower accuracy of the older version of NetMHCpan software which was used in the Rao et al. study (v2.0, released in 2009 and trained only on *in vitro* binding affinity data, while in v4.0, used in the current study, MS eluted ligand data is also incorporated).

Thorough investigation of HLA presentation biases can lead to better understanding of mechanisms underlying the existence of both beneficial HLA alleles and those alleles leading to disease susceptibility in various scenarios ranging from infectious diseases to autoimmunity. The COVID-19 pandemic has highlighted the necessity of a rapid selection of vaccine targets. HLA binding preferences should evidently be taken into account together with the population frequency of HLA alleles during vaccine development. We hope that our findings can help to explain why certain HLAs are more likely to present peptides from specific viral proteins compared to others. Those presentation biases may arise due to evolutionary fine-tuning of the HLA presentation machinery optimizing selection of non-self peptides, a subject for future studies.

## Data availability statement

The datasets presented in this study can be found in online repositories. The names of the repository/repositories and accession number(s) can be found below: https://github.com/antigenomics/hla-go-ms, GitHub, user:antigenomics, repository:hla-go-ms.

## Author contributions

Experimental data: WP, TP, AN, SB, NT and PB. Data analysis: VK, WP, DS, IBW, MS. Manuscript draft and editing: MS, VK, IW, IZ, DC, HK, WP, PB. Supervised the study: MS, IZ, DC, HK, PB. All authors contributed to the article and approved the submitted version.

## Acknowledgements

This work was supported by RFBR grant No. 19-015-00551 (IZ), Ministry of Science and Higher Education of the Russian Federation grant No. 075-15-2019-1789 (MS, DMC and DS), Medical Research Council (MRC) programme grant MR/K012037 (PB) and National Institutes of Health grant R01 AI 118549 (PB). PB is a Jenner Institute Investigator. HK is funded by MRC Human Immunology Unit core funding. We would like to thank Masafumi Takaguchi for providing the CD4.721.221 cell line, Vasily Tsvetkov and Ekaterina Putintseva for their assistance with running HLA binding prediction algorithms.

## Conflict of interest

The authors declare that the research was conducted in the absence of any commercial or financial relationships that could be construed as a potential conflict of interest.

## Publisher’s note

All claims expressed in this article are solely those of the authors and do not necessarily represent those of their affiliated organizations, or those of the publisher, the editors and the reviewers. Any product that may be evaluated in this article, or claim that may be made by its manufacturer, is not guaranteed or endorsed by the publisher.
