# Supplementary material for: HLA variants have different preferences to present proteins with specific molecular functions which are complemented in frequent haplotypes
Source: Front Immunol. 2022 Dec 20;13:1067463. doi: 10.3389/fimmu.2022.1067463 (PMC9808399; doi:10.3389/fimmu.2022.1067463)
Supplement: Supplementary file 1 [file DataSheet_1.pdf]

Supplementary Figures and Tables

Supplementary Table S1. Extended set of 93 HLA alleles used for validation of the conclusions from a smaller set. The list and frequencies of the alleles covering 95% of individuals worldwide were taken from Sarkizova et al. [https://www.nature.com/articles/s41587-019-0322-9#Sec33]. Anchor residues in the second (P2) and the ninth (P9) positions were inferred if the aggregated fraction of one of the groups of amino acids with similar physicochemical properties was greater than 0.5 for the corresponding position of netMHCpan binders for that allele.

Allele	Freq.	P2 anchor	P9 anchor
HLA-A0101	0.048	ST	
HLA-A0201	0.153	LFIMV	LFIMV
HLA-A0202	0.008	LFIMV	LFIMV
HLA-A0203	0.015	LFIMV	LFIMV
HLA-A0204	0.003	LFIMV	LFIMV
HLA-A0205	0.008	LFIMV	LFIMV
HLA-A0206	0.035	LFIMV	LFIMV
HLA-A0207	0.021	LFIMV	LFIMV
HLA-A0211	0.003	LFIMV	LFIMV
HLA-A0301	0.043	LFIMV	RK
HLA-A1101	0.117		RK
HLA-A1102	0.007		RK
HLA-A2301	0.023		LFIMV
HLA-A2402	0.188		LFIMV
HLA-A2407	0.005		LFIMV
HLA-A2501	0.005		LFIMV
HLA-A2601	0.034		LFIMV
HLA-A2902	0.016		Y
HLA-A3001	0.025		
HLA-A3002	0.015		Y
HLA-A3101	0.041		RK
HLA-A3201	0.014	LFIMV	LFIMV
HLA-A3301	0.012		RK
HLA-A3303	0.041		RK
HLA-A3401	0.016		RK
HLA-A3402	0.005		RK
HLA-A3601	0.004		Y
HLA-A6601	0.006		
HLA-A6801	0.023		RK
HLA-A6802	0.013		LFIMV
HLA-A7401	0.008	LFIMV	RK

Allele	Freq.	P2 anchor	P9 anchor
HLA-B0702	0.041	P	LFIMV
HLA-B0704	0.031	P	LFIMV
HLA-B0801	0.03		LFIMV
HLA-B1301	0.026		LFIMV
HLA-B1302	0.014		LFIMV
HLA-B1402	0.012		LFIMV
HLA-B1501	0.034		LFIMV
HLA-B1502	0.013		LFIMV
HLA-B1503	0.012		LFIMV
HLA-B1510	0.006		LFIMV
HLA-B1517	0.003	ST	LFIMV
HLA-B1801	0.023	DE	
HLA-B2705	0.012	RK	
HLA-B3501	0.055		LFIMV
HLA-B3503	0.009		LFIMV
HLA-B3507	0.013		
HLA-B3701	0.025	DE	LFIMV
HLA-B3801	0.008		LFIMV
HLA-B3802	0.009		LFIMV
HLA-B4001	0.051	DE	LFIMV
HLA-B4002	0.042	DE	LFIMV
HLA-B4006	0.018	DE	LFIMV
HLA-B4201	0.01	P	LFIMV
HLA-B4402	0.022	DE	LFIMV
HLA-B4403	0.045	DE	LFIMV
HLA-B4501	0.01	DE	
HLA-B4601	0.024		LFIMV
HLA-B4901	0.009	DE	LFIMV
HLA-B5001	0.009	DE	
HLA-B5101	0.052		LFIMV
HLA-B5201	0.023		LFIMV
HLA-B5301	0.016		LFIMV
HLA-B5401	0.014	P	
HLA-B5501	0.006	P	
HLA-B5502	0.01	P	
HLA-B5601	0.014	P	
HLA-B5701	0.01	ST	LFIMV

Allele	Freq.	P2 anchor	P9 anchor
HLA-C0102	0.085		LFIMV
HLA-C0202	0.028		LFIMV
HLA-C0302	0.025		LFIMV
HLA-C0303	0.056		LFIMV
HLA-C0304	0.091		LFIMV
HLA-C0401	0.112		LFIMV
HLA-C0403	0.019		LFIMV
HLA-C0501	0.026		LFIMV
HLA-C0602	0.062		LFIMV
HLA-C0701	0.069		LFIMV
HLA-C0702	0.131		LFIMV
HLA-C0704	0.015		LFIMV
HLA-C0801	0.045		LFIMV
HLA-C0802	0.02		LFIMV
HLA-C1202	0.032		LFIMV
HLA-C1203	0.02		LFIMV
HLA-C1402	0.025		LFIMV
HLA-C1403	0.015		LFIMV
HLA-C1502	0.034		LFIMV
HLA-C1601	0.024		LFIMV
HLA-C1701	0.019		LFIMV
HLA-C1801	0.006		LFIMV

					HLA-B5703	0.005	ST	LFIMV					
					HLA-B5801	0.029	ST	LFIMV					
					HLA-B5802	0.008	ST	LFIMV					

Supplementary Table S2. Populations included in the analysis of HLA presentation bias compensation in haplotypes. Haplotypes and their frequencies were taken from http://allelefrequencies.net/.

Population	Sample size	Number of haplotypes
USA NMDP Caribbean Indian	14,339	89
USA NMDP American Indian South or Central America	5,926	65
USA NMDP Hawaiian or other Pacific Islander	11,499	63
USA NMDP Filipino	50,614	51
USA NMDP Hispanic South or Central American	146,714	45
USA NMDP Chinese	99,672	44
USA NMDP Caribbean Hispanic	115,374	42
USA NMDP Mexican or Chicano	261,235	42
USA NMDP Southeast Asian	27,978	39
USA NMDP Japanese	24,582	38
USA NMDP Korean	77,584	37
USA NMDP North American Amerindian	35,791	35
USA NMDP Caribbean Black	33,328	34
USA NMDP Vietnamese	43,540	34
USA NMDP Middle Eastern or North Coast of Africa	70,890	33
USA NMDP African American pop 2	416,581	29
USA NMDP European Caucasian	1,242,890	29
USA NMDP South Asian Indian	185,391	29
USA NMDP African	28,557	28

Supplementary Table S3. Number of predicted 9-mer HLA ligands, HLA ligand enriched (HLEP) and depleted (HLDP) proteins for every HLA allele used in this study.

Allele	Number of enriched genes	Number of depleted genes	Ligand count
HLA-A*02:01	1946	1557	655638
HLA-A*11:01	55	106	498485
HLA-B*07:02	614	220	365507
HLA-B*27:05	671	239	333662

HLA-C*02:02	1015	770	657146
HLA-C*15:02	964	500	553180

Supplementary Table S4. Number of predicted HLA-A*11:01 ligands, HLA ligand enriched (HLEP) and depleted (HLDP) proteins for 8-12mer peptides.

Ligand length	Number of enriched genes	Number of depleted genes	Ligand count
8	56	25	89334
9	55	106	498485
10	108	233	676915
11	306	295	354065
12	362	443	422447

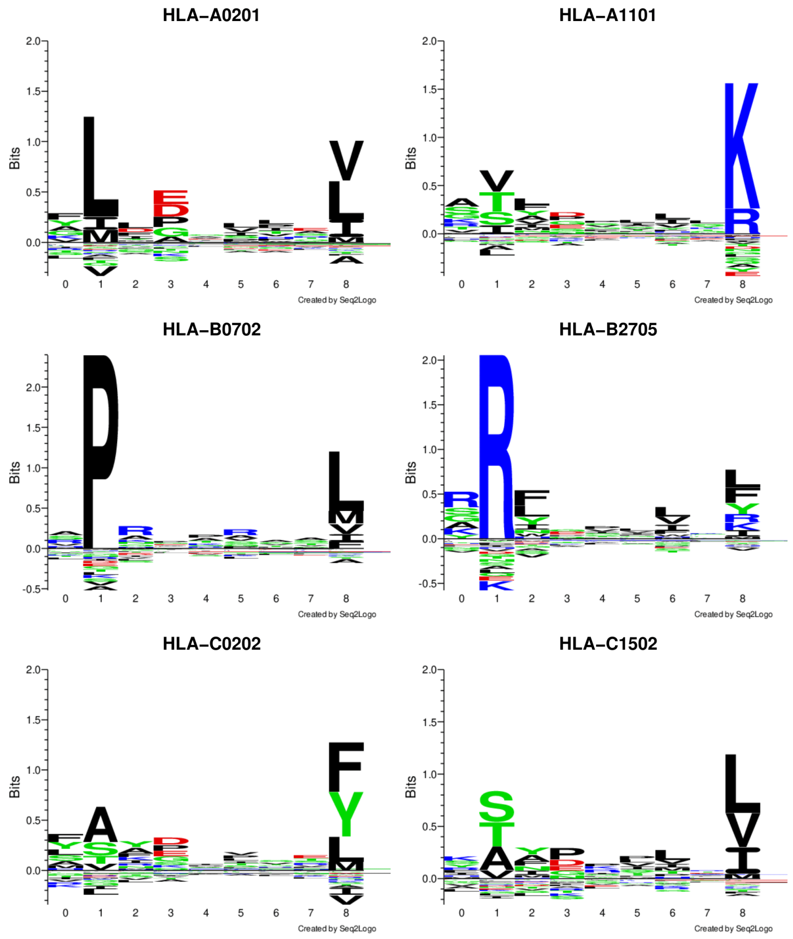

Supplementary Figure S1. Binding motifs for surveyed HLA alleles. Motifs for naturally presented HLA ligands for a sample set of alleles used in the study (data from http://www.cbs.dtu.dk/services/NetMHCpan/logos_ps.php). The height of the bar is equal to the information content at each amino acid position. Enriched amino acids are displayed on the positive y-axis, depleted amino acids are displayed on the negative y-axis, the relative height of each amino acid is proportional to the log-odds ratio. Physicochemical characteristics of amino acids are color coded: black, hydrophobic; green, neutral polar; blue, basic; red, acidic.

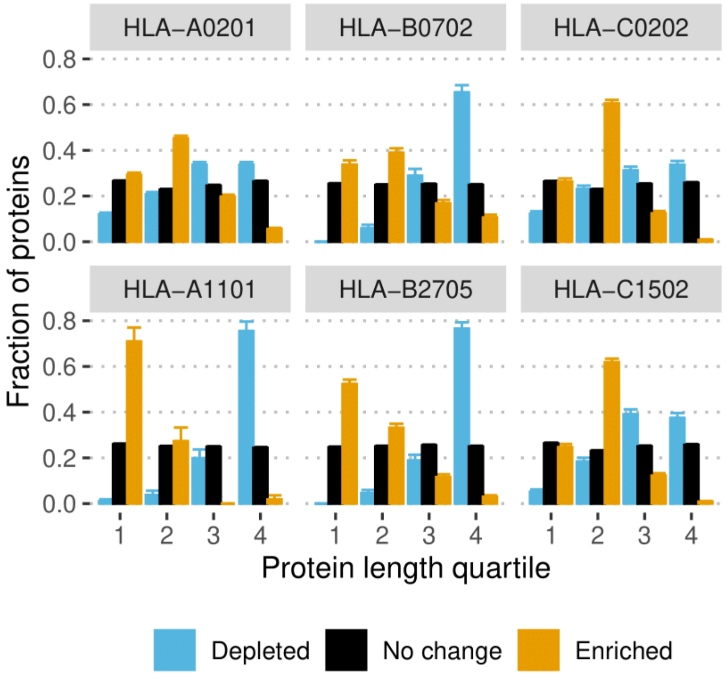

Supplementary Figure S2. Length bias for proteins enriched (HLEPs) and depleted (HLDPs) in HLA ligands. Plot shows the fraction of genes expressing proteins residing in four gene quartiles (from 1 - shortest to 4 - longest) in HLEP (Enriched) and HLDP (Depleted) sets compared to remaining proteins (No change). Error bars show 95% confidence interval for fractions. Note that genes enriched in HLA ligands are shifted towards shorter length while depleted ones are shifted towards longer lengths.

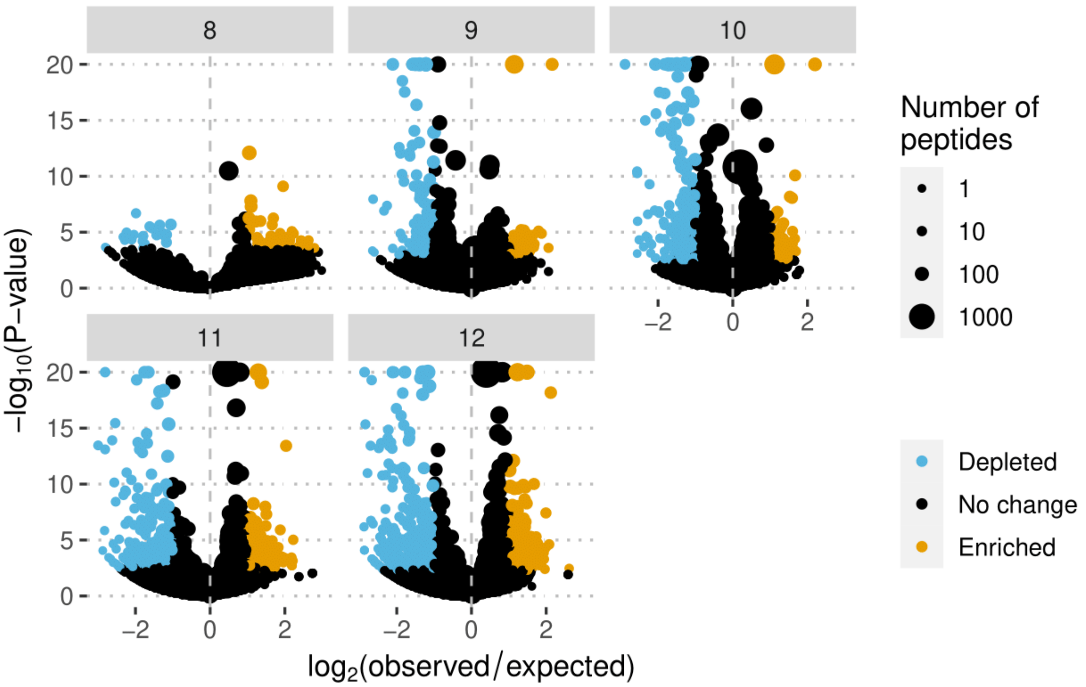

Supplementary Figure S3. Ligand enrichment analysis for HLA-A*11:01 allele and different ligand lengths. Volcano plots showing the log of the ratio of observed and expected HLA-A*11:01 ligands for each human gene plotted against enrichment P-value computed using binomial test. Point size shows number of predicted HLA ligands, point color highlights genes enriched and depleted in ligands according to 2 times odd differences and adjusted P-value of < 0.05. Data for different peptide lengths (8, 9, 10, 11 and 12 amino acids) are shown as separate plots.

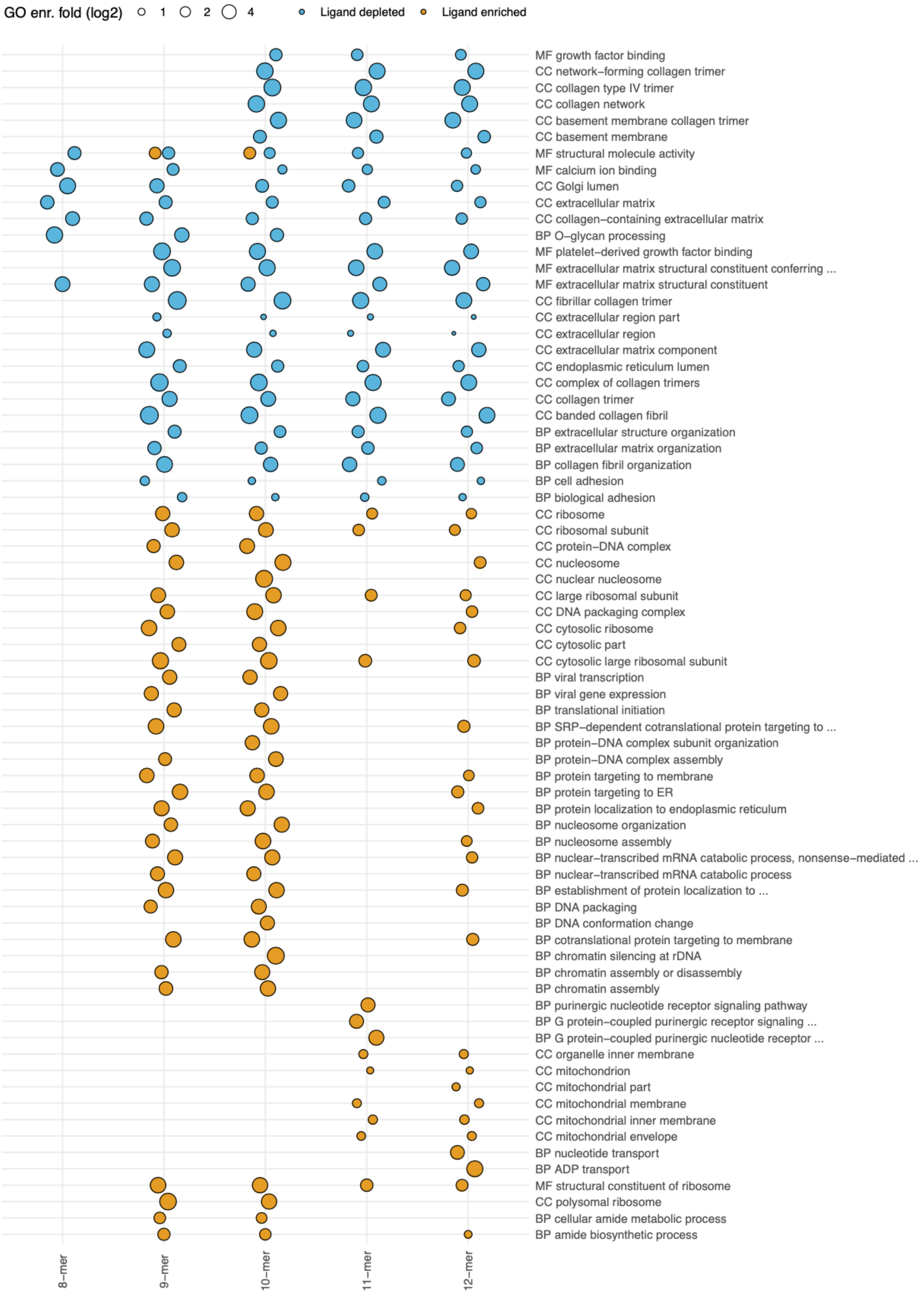

Supplementary Figure S4. Gene ontology (GO) term enrichment analysis for human genes differentially enriched in ligands of various lengths by HLA-A*11:01. Point size represents the GO enrichment fold for genes enriched (yellow) and depleted (blue) in HLA 8- to 12-mer HLA ligands. An adjusted P-value threshold of 0.01 was chosen and top 20 GO categories were chosen for each HLA allele.

A

Ontology	Term	Alleles
MF	structural molecule activity	89 (96%)
CC	supramolecular complex	86 (92%)
CC	supramolecular polymer	86 (92%)
MF	extracellular matrix structural constituent	84 (90%)
CC	collagen trimer	84 (90%)
CC	fibrillar collagen trimer	84 (90%)
MF	extracellular matrix structural constituent conferring tensile strength	84 (90%)
CC	banded collagen fibril	84 (90%)
CC	complex of collagen trimers	83 (89%)
MF	platelet–derived growth factor binding	82 (88%)
CC	collagen type IV trimer	81 (87%)
BP	extracellular matrix organization	81 (87%)
BP	extracellular structure organization	81 (87%)
CC	network–forming collagen trimer	81 (87%)
CC	collagen network	81 (87%)
CC	basement membrane collagen trimer	81 (87%)
CC	extracellular matrix	80 (86%)
BP	collagen–activated tyrosine kinase receptor signaling pathway	80 (86%)
CC	collagen–containing extracellular matrix	80 (86%)
CC	endoplasmic reticulum lumen	76 (82%)

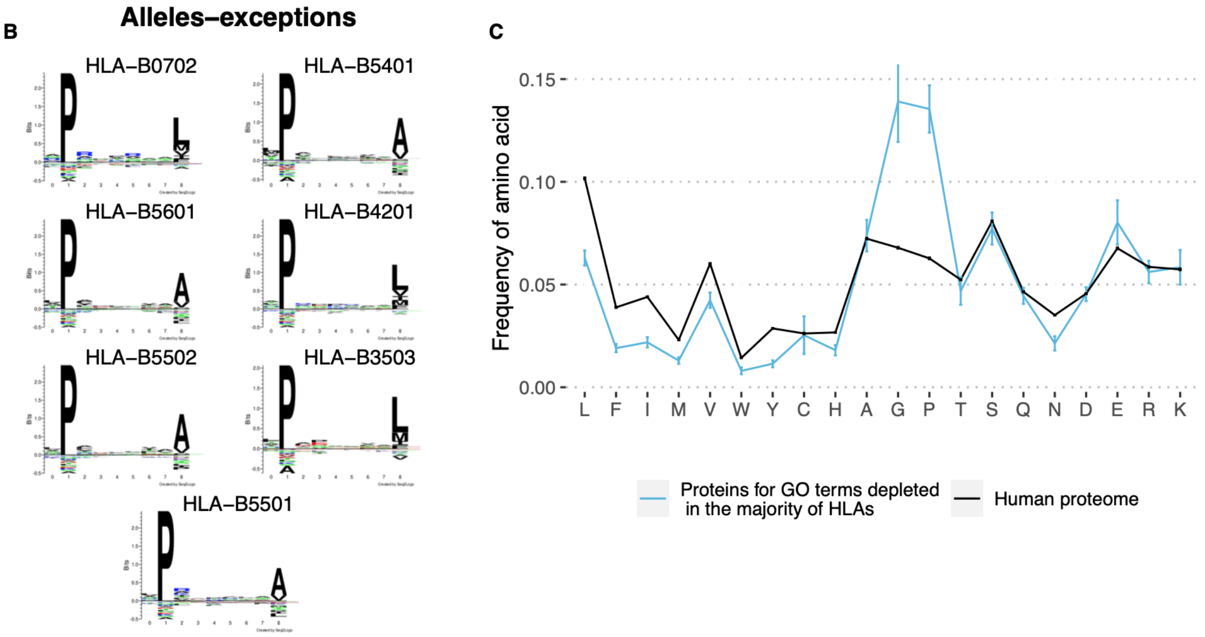

Supplementary Figure S5. GO categories associated with HLDPs for most alleles in the extended dataset. A. GO terms corresponding to HLDPs for more than 80% of the surveyed 93 HLA alleles. **B.** Motifs of naturally presented ligands for alleles-exceptions in which less than 15% of GO terms from table **A.** are depleted. Note that all these alleles require proline as anchor residue in P2. For all the alleles without proline anchors at least 70% of GO terms from table **A.** are depleted. **C.** Comparison of amino acid composition of proteins corresponding to GO categories from **A.** (consider only proteins which are in HLDPs for at least half of surveyed 93 alleles but not for any of the list from **B.**) and human proteome. Error bars show 95% confidence interval for the mean value. Note that glycines and prolines are enriched in these proteins as in **Figure 3C**.

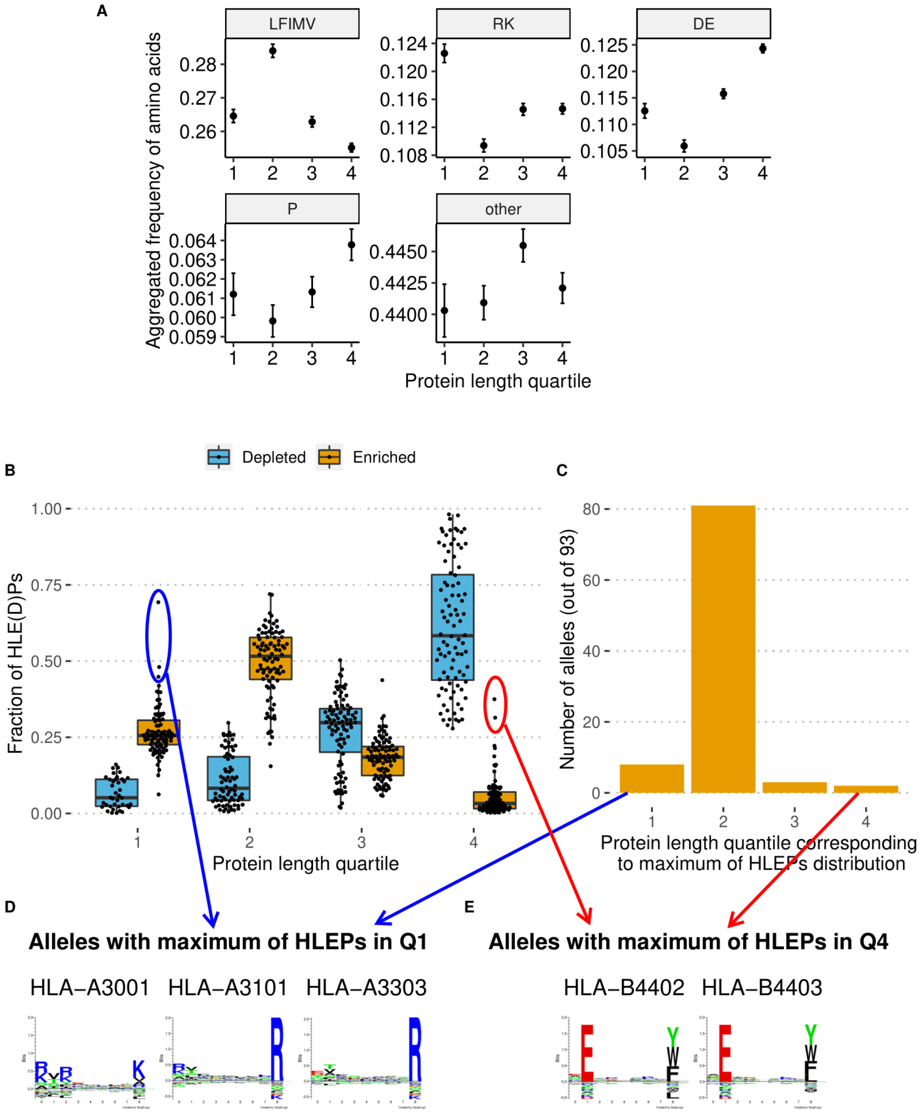

Supplementary Figure S6. Analysis of length bias of HLE(D)Ps on an extended dataset from 93 HLA alleles. A. Fractions of amino acids in proteins of different length quartiles. Amino acids are grouped as the following: “LFIMV” stands for hydrophobic amino acids (leucine, phenylalanine, isoleucine, methionine, and valine), “DE” - negatively charged amino acids (aspartic acid and glutamic acid), “RK” - positively charged amino acids (arginine and lysine), P - proline, “other” - the remaining 10 amino acids which are rarely used as anchor residues. Note that the highest fractions of hydrophobic, positively and negatively charged amino acids are for proteins from Q2, Q1, and Q4, respectively. Error bars show 95% confidence interval for the mean value. **B.** Distribution of the length (split to quartiles) of HLE(D)Ps for the surveyed 93 HLA alleles. Note that on average this distribution peaks in Q2 for HLEPs and in Q4 for HLDPs. **C.** Number of alleles for which HLEPs length distributions profiles from **B.** have maximums in Q1, Q2, Q3, and Q4. Note that for the majority of alleles (80 out of 93) the highest fraction of HLEPs correspond to Q2 proteins, while some alleles have preferences for presentation of proteins from Q1, Q3, or Q4. **D-E**. Motifs of naturally presented ligands for alleles, which have distributions of HLEPs with the maximum on Q1 (**D**) or Q4 (**E**). Note that “Q1-max” alleles require positively charged anchor residues which are enriched in Q1 proteins (see **A.**) while “Q4-max” ones require negatively charged anchor residues which are enriched in Q4 proteins. Motifs are taken from http://www.cbs.dtu.dk/services/NetMHCpan/logos_ps.php.

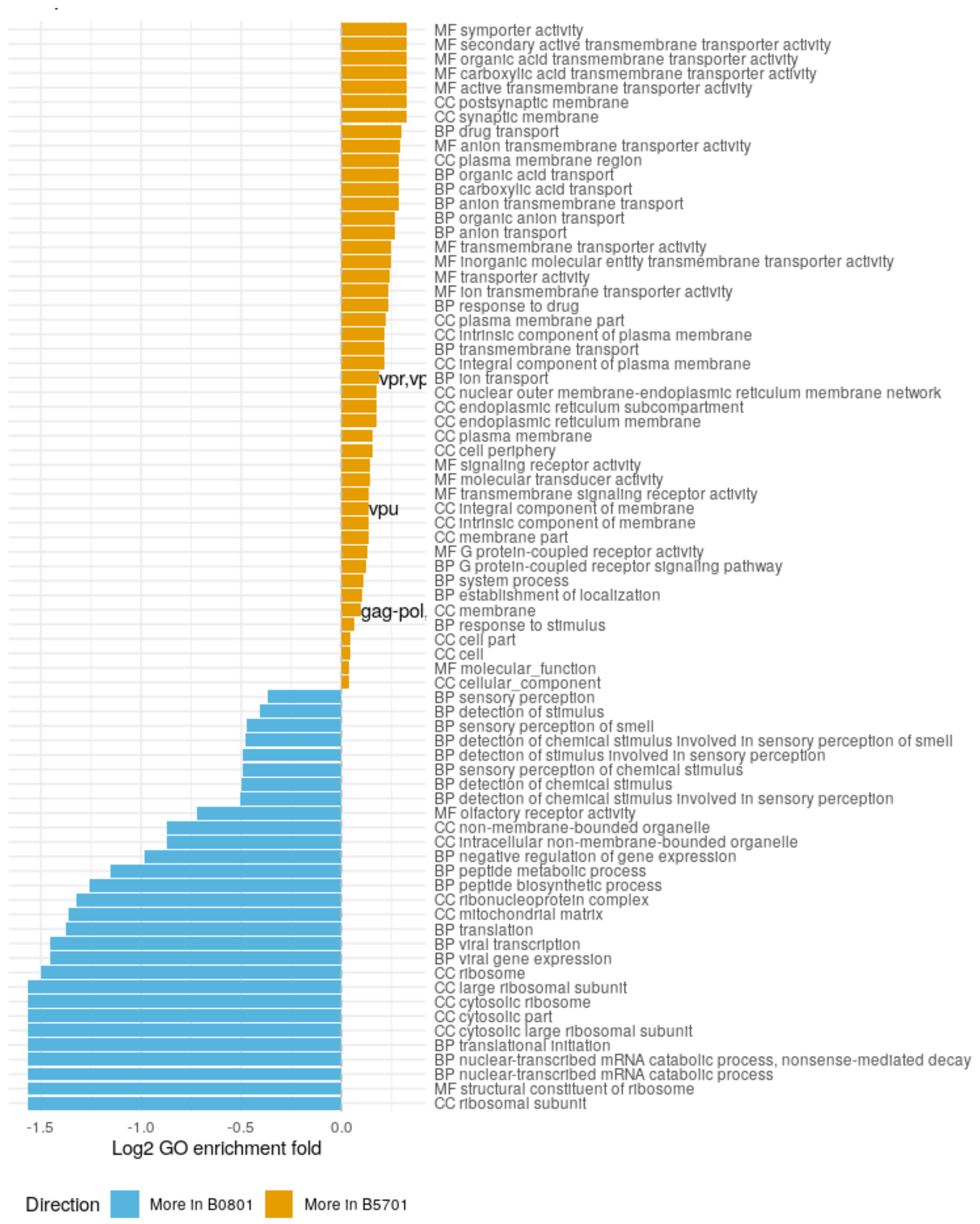

Supplementary Figure S7. Analysis of GO categories enriched for HIV-protective and non-protective HLA alleles. Bar plot shows GO category enrichment fold for HLA-B*57:01 (HIV-protective, yellow) and HLA-B*08:01 (non-protective, blue) HLA alleles. Only categories with an adjusted enrichment P-value less than 0.01 are shown, P-values were computed in a GO enrichment test where ligand-enriched genes for HLA-B*57:01 were used as a test set and HLA-B*08:01-ligand-enriched genes were used as control and vise versa. HIV protein names corresponding to GO categories are shown according to data from UniProt/QuickGO.
